# How to minimize children’s environmental tobacco smoke exposure: an intervention in a clinical setting in high risk areas

**DOI:** 10.1186/1471-2431-13-76

**Published:** 2013-05-15

**Authors:** Noomi Carlsson, AnnaKarin Johansson, Agneta Abrahamsson, Boel Andersson Gäre

**Affiliations:** 1 Department of Clinical and Experimental Medicine, Division of Paediatrics, Faculty of Health Sciences, Linköping University, SE-581 83, Linköping, Sweden; 2 Department of Public Health and Medical Care, Jönköping County Council, Box 1024, SE-551 11 Jönköping, Sweden; 3 Department of Medicine and Health, Division of Nursing Science, Faculty of Health Sciences, Linköping University, SE-581 83, Linköping, Sweden; 4 Department of Health and Society, University College of Kristianstad, SE-291 88, Kristianstad, Sweden; 5 Futurum – the Academy for Healthcare, Jönköping County Council, SE-551 85, Jönköping, Sweden; 6 The Jönköping Academy for Improvement of Health and Welfare, Jönköping University, Box 1026, SE-551 11 Jönköping,Sweden

**Keywords:** Children, Child Health Care, Tobacco smoke prevention, Passive smoking

## Abstract

**Background:**

Despite the low prevalence of daily smokers in Sweden, children are still being exposed to environmental tobacco smoke (ETS), primarily by their smoking parents. A prospective intervention study using methods from Quality Improvement was performed in Child Health Care (CHC). The aim was to provide nurses with new methods for motivating and supporting parents in their efforts to protect children from ETS exposure.

**Method:**

Collaborative learning was used to implement and test an intervention bundle. Twenty-two CHC nurses recruited 86 families with small children which had at least one smoking parent. Using a bundle of interventions, nurses met and had dialogues with the parents over a one-year period. A detailed questionnaire on cigarette consumption and smoking policies in the home was answered by the parents at the beginning and at the end of the intervention, when children also took urine tests to determine cotinine levels.

**Results:**

Seventy-two families completed the study. Ten parents (11%) quit smoking. Thirty-two families (44%) decreased their cigarette consumption. Forty-five families (63%) were outdoor smokers at follow up. The proportion of children with urinary cotinine values of >6 ng/ml had decreased.

**Conclusion:**

The intensified tobacco prevention in CHC improved smoking parents’ ability to protect their children from ETS exposure.

## Background

Children’s exposure to tobacco smoke is primarily attributable to their smoking parents. Smoking parents often use different strategies to minimize children’s exposure to environmental tobacco smoke (ETS) [1-3].

A number of studies around the world have measured the impact of various interventions intended to influence parents’ smoking behaviour [4]. In a review of these interventions by Priest et al. the conclusion was that there is insufficient evidence to support one strategy over another. However, some recent studies designed to apply smoking restrictions in the home have proved successful even if parents do not quit smoking [5,6]. One problem with health promoting interventions is to make them sustainable by the professionals in clinical settings. Another problem is that programs are rarely followed in their entirety by health care professionals and are therefore at risk of not achieving the desired effects [7,8].

Several studies show that children’s ETS exposure is related to the socioeconomic situation of their parents [9,10] and the parent’s country of birth [11]. Several dimensions of socioeconomic positions have to be considered in explanations of social inequalities in families’ home smoking practices and hence children’s ETS exposure [12,13]. Swedish studies have shown that selective actions to reach these families are not carried out by CHC nurses [14], they experience difficulties in contact with foreign born parents and they miss support in their mission [15].

The prevalence of daily smokers in Sweden decreased from 16% in 2004 to 11% in 2011, but there are differences seen between socio-economic groups. Daily smoking is five times more common among people with a lower level of education than among those with a higher level of education [16]. This can be one contributing factor to inequalities in health, since children with smokers in their families are at risk of adverse health effects both during their childhood and as adults [17,18]. The prevalence of smokers in families with children born in 2009 in Sweden was 13.2% when the children were 0 to 4 weeks of age and13.6% when the children were 8 months [19]. Two earlier studies have found that neither professionals nor parents were satisfied with the tobacco preventive work carried out in CHCs. These studies also found that fathers, foreign born parents and socio-economically disadvantaged groups were perceived as more difficult to reach than other groups [20,21]. Thus, there is a need to develop new strategies and methods to reach these groups in order to minimize ETS exposure in small children.

Several studies have pointed out that systematic implementation of evidence-based methods is often absent, slow or variable, a factor which may lead to inequality in care and researchers have stated “there is a gap between what we know and what we do” [22,23]. Similar patterns, showing wide variations in both process and result measures between different Swedish counties, have been found in national comparison of preventive work and healthcare [24]. Methods from Continuous Quality Improvement (CQI) can be helpful in lowering the unwanted variation and thus decrease inequalities in care and outcomes [22,25]. Quality Improvement (QI) methods such as “collaborative learning” have been used in health care resulting in improved clinical results [26,27]. To our knowledge this methodology has not been applied in tobacco preventive work in Child Health Care in Sweden.

The aim of this study was to design and evaluate an improvement project comprising a “bundle” of actions in CHCs with the aim of protecting children from ETS exposure. The intervention was directed towards smoking parents in areas where children had a high risk of ETS exposure.

## Methods and study population

### Selection of CHC areas

The inclusion criteria for CHC centres was that they served areas with a high proportion of smoking parents (>10% of smokers in families of 8 month old children), according to information derived from CHC’s annual statistics. The “high risk areas” were further characterized by a high proportion of foreign born parents (born outside Sweden), a high proportion of adults with a lower level of education (≤12 years) and a high proportion of families receiving social welfare benefits. This information was retrieved from Statistics Sweden in 2009 that linked the various postal codes for these areas with the characteristics.

Another prerequisite for inclusion was that the nurses at CHC centres were trained in Motivational Interviewing (MI) [28] and that all CHC nurses had the possibility to give parents a referral to a certified tobacco treatment specialist [29]. Twenty-nine eligible areas were invited and fifteen areas were represented in the intervention.

### Recruitment of CHC nurses

Sixty-five nurses working in thirty-seven areas which fulfilled the criteria for inclusion were sent an e-mail with information about the study and an invitation to participate. One of the authors had personal meetings with the nurses to provide in-depth information about the study and answer questions. Twenty-four nurses from 15 different CHC centres serving both urban/suburban and rural areas agreed to participate. Reasons given for not participating were; taking part in other projects, retirement, and lack of time during the period of the intervention. Two of the nurses who chose to participate changed jobs within one month of the start of the study and thus discontinued participation.

### Recruitment of parents

**Figure 1:**
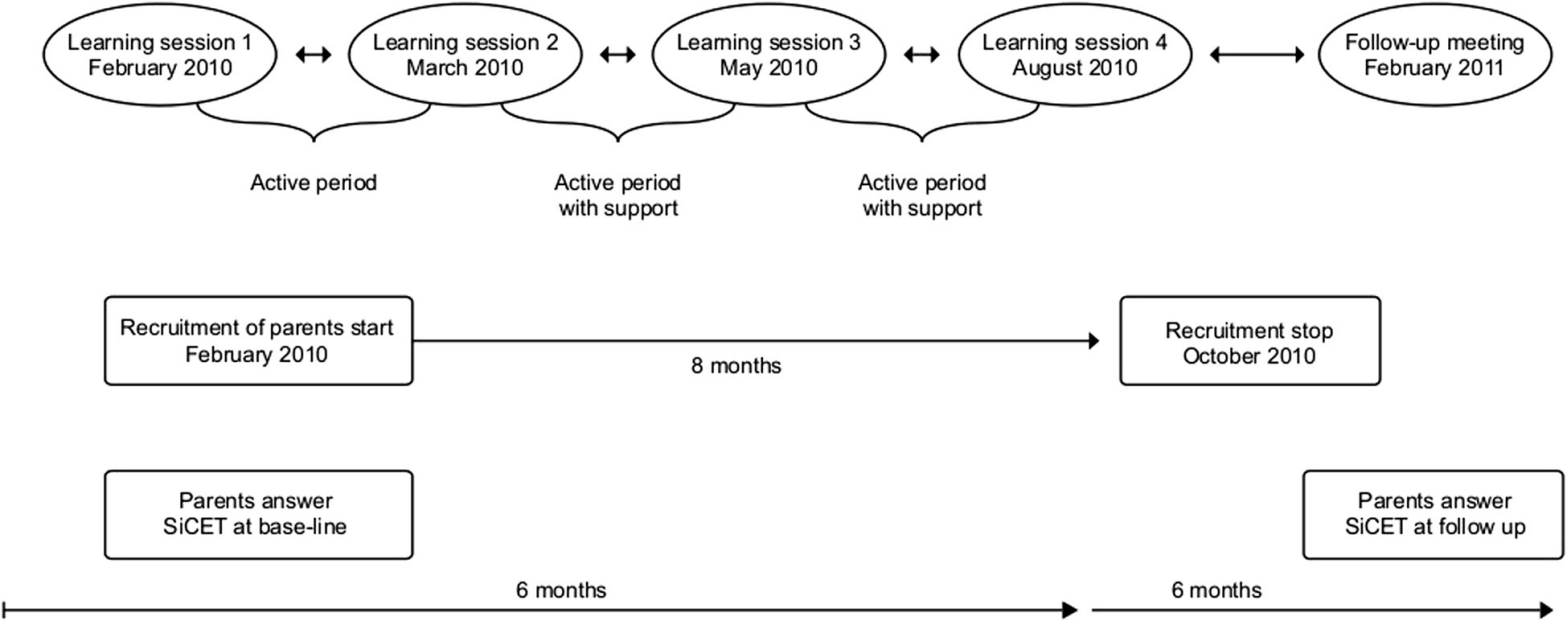
**The improvement work was designed according to a “collaborative learning model” for child health care nurses (The Institute for Healthcare Improvement, Kilo 1998; **[25]**).** Nurses recruited families during the 8 month intervention period. The SiCET was answered at base-line, in some cases 8 months after inclusion in the study and at follow up, 12 months after inclusion. Nurses worked actively with the parents between learning sessions and had extra support from one of the authors (NC) during the active periods. A follow-up meeting was held 6 months after learning session 4.

During the active periods between learning sessions (Figure 1) the nurses were asked to invite families with new-borns or children under the age of five years who had a smoking family member in the home to participate in the study. The nurses invited 124 families. Eighty-six families agreed to participate, and 72 of them fulfilled the whole intervention period.

### Improvement project design

#### The intervention

The intervention was conducted between February 2010 and October 2011 in a county in south-eastern Sweden. The intervention was carried out with an interactive approach where the researcher was given access to an understanding based on the participants’ own perspectives, both from the CHC nurses and the parents [30]. The results from the two earlier studies [20,21] formed the basis for the intervention which was designed to reduce children’s ETS exposure in their homes in “high risk areas”. In order to successfully communicate with parents with different backgrounds, the dialogue between nurses and parents needed to be improved, and in order to make this improvement, different evidence based components were combined in an “intervention bundle”.

#### The components of the intervention bundle

The “bundle” [31] was built on evidence based methods presented in Table 1. The nurses were encouraged to use their skills in Motivational Interviewing (MI) [32], and were also taught how to use and were expected to use the Smoking in Children’s Environment Test (SiCET) to help facilitate dialogue with all parents. The SiCET is a questionnaire which is supposed to be answered by the parents and then used to facilitate the dialogue between nurses and parents. It includes questions on tobacco consumption, where smoking is performed in the home, and how prepared parents consider themselves to be to make changes. The questionnaire is a validated instrument, developed and tested to measure children’s ETS exposure [33], and evaluated for use as a facilitator in the dialogue between parents and CHC nurses [34].

**Table 1:** The table summarizes “the bundle”, which includes the actions the nurses were supposed to use in the intervention

**Activity**	Reference*
Collaborate with Antenatal Care	Facilitate parental support. (39, 40)
Home visits	Support in home environment. (41)
Use of Smoking in Children’s Environment Test (SiCET)	Investigate children’s ETS exposure. A basis for dialogue. (33, 34)
Introduce websites	Support in quitting smoking from websites. (35)
Introduce booklets	Information to parents. (37, 38)
Invite and use interpreter	Facilitating the dialogue with parents in their native language. (58, 59)
Motivational Interviewing (MI)	A positive approach to motivating parents’ behaviour changes. (28, 32)
Referrals to an expert in smoking cessation support	Parents have the possibility to meet a certified tobacco treatment specialist. (29)

*References showing the evidence for using these actions are shown in the right column.

Nurses were also encouraged to use and recommend Swedish smoking cessation websites such as ‘quit-smoking-line’ [35], the booklet ‘Tobacco-free children’ (developed by the Swedish National Institute of Public Health [36]) and other written material in order to support the parents [37,38] in their ambitions to stop smoking or change their smoking behaviour. Written information was made available in the nine different languages used by participating parents. Additional actions recommended in the intervention were; cooperation with antenatal care [39,40] and social services, home-visits [41], the use of interpreters, and referrals to an expert in smoking cessation support. In the regular program the nurses meet the families approximately 15 times during the child’s first year and have good opportunities to have dialogues about smoking with parents and follow their steps in the changing process. In the study each nurse was expected to plan how and when the components in the bundle were appropriate to be introduced to the family. The nurses were also asked to register their own efforts and the parents’ actions in log-books, using one for each family.

#### The method for implementation – “Collaborative learning”

The method used for implementation and learning in this study was the QI approach often referred to as “collaborative learning” [42,43]. This concept is based on bringing groups with a common aim together to adapt, spread and test knowledge and good ideas in practice [25,27,44]. The collaborative included four learning sessions over a 6 months period and a follow-up meeting after one year (Figure 1). A project management group was set up comprising a pediatrician in charge of child health services, an expert in tobacco use, a professional in smoking cessation support, a social-anthropologist, an epidemiologist and a QI project management expert. The group met during the planning stage before the intervention and was consulted when appropriate during the period of the collaborative when support and advice was needed by the author who led the nurses’ learning sessions [25].

The nurses’ learning sessions covered the following subject areas; health risks associated with children’s ETS exposure, other smoking related issues, smoking cessation support, how to use interpreters, and communication skills. In addition, nurses were introduced to QI methods and had time for group discussions and to prepare tests at CHC centres according to the Model for Improvement with Plan-Do-Study-Act (PDSA) cycles [25]. In this methodology the improvers (in this case the nurses), in their local context, ask and act on the following questions: What are we trying to accomplish? How do we know a change is an improvement? What changes will result in improvement?

One effect of the interactive approach in the design of the intervention was the development of a checklist by the nurses during the learning sessions. The checklist became a support tool for the nurses in their improvement work at their CHC.

#### Methods used in the evaluation of the improvement project

In the study of the effects of the improvement project three sets of data were used 1) data from the SiCET questionnaires, 2) urine cotinine analyses in children’s urine and 3) data from the nurses’ log-books.

1. The SiCET

The results from the SiCET questionnaire were used to evaluate the effects of the improvement project. The questionnaire was answered by the parents when they were included in the study and at follow up one year later. Some parents also answered the SiCET on an additional occasion during the intervention.

2. Cotinine levels in children’s urine

Cotinine is a metabolite of nicotine which can be detected in blood, urine and saliva. The aims of measuring cotinine levels in children’s urine were 1) to compare the child’s ETS exposure to their parent’s description of their smoking habits in the SiCET [2], and 2) to compare children’s cotinine levels <6 ng/ml (=lower level of quantification) and levels >6 ng/ml at base-line and follow up. A typical value for cotinine levels in urine in a person exposed to ETS is 6 ng/ml which corresponds to a daily nicotine intake of 80 μg. An appropriate cut-off point for urinary cotinine discriminating active smokers from non-smokers is assumed to be 60 μg/L [45]. Despite some methodological weaknesses, e.g. individual differences in the metabolism of nicotine and the relatively short half-time (approximately 20 hours), it has been regarded as the best available biomarker of ETS exposure today [45]. In this study each child was compared with itself.

The urine samples were provided by children during CHC visits or home visits. No smoking was performed while the sample was taken. The samples were cooled and transported to the Biobank at Ryhov Hospital, Jönköping, where they were frozen. The samples were then stored at -20°C and transported to McNeil, Helsingborg, to be analysed. Cotinine analyses were performed with Gas Chromatography, Internal method NM-427-3. The method is validated for cotinine > 6 ng/ml, lowest level of quantification (LLOQ). The first urine test was taken at inclusion in the study and the second after one year. Parents were informed if cotinine levels found in their children’s urine were below or above the measureable limit (6 ng/ml).

3. Log-books

The nurses’ log-books were designed by the research team and included one column for actions carried out by nurses and some space for free text. The log-books provided information on the number of meetings the parents and the nurse had during the intervention. All data in the log-books were coded into numbers in order to be analysed along with other data.

### Statistics

Data analyses were carried out using the statistical software SPSS (Statistical Package for the Social Sciences, version 20) and SAS 9.2. Descriptive statistics were used to present the results, and Chi-square test and Fisher’s Exacta test were used to analyse non-parametric data. Differences between groups were calculated using Student’s t-test for normally distributed variables. The Wilcoxon Signed Ranks Test was used to determine the differences between the first and second urine test due to the cotinine levels not being normally distributed. Logistic regression was used to analyse if there were any relation between nurses’ activities (independent variables) and the outcome of the SiCET at follow up (dependent variable), adjusted for parent’s backgrounds variables. The outcome of the SiCET was dichotomised into outdoor or indoor smokers. Outdoor smokers were defined as strict smoking outdoors with the door closed with or without changing clothes afterwards. A p-value <0.05 was regarded as significant.

### Ethical issues

The study was approved by the research ethics committee in Linköping, Sweden (application registration number: Dnr M114-07). Written informed consent for participation in the study was obtained from all parents. Nurse’s participation was approved by the managers of the nurses. The nurses were informed orally and in writing including that their participation was voluntary and that they could discontinue participation at any time without any explanation. All data collected would be treated confidentially.

## Results

**Figure 2:**
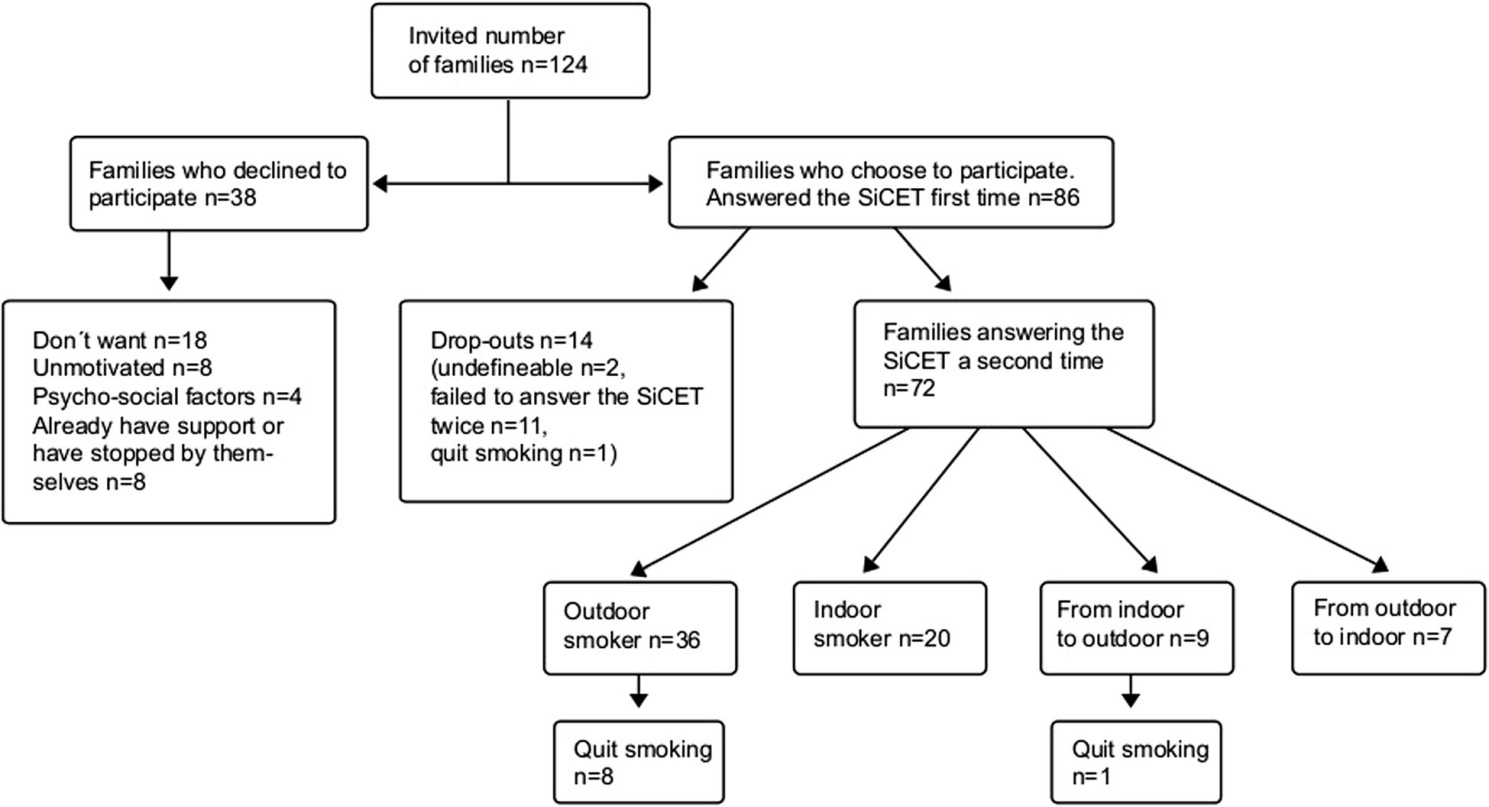
**Number of families who were asked to participate in the study, families who declined and their reasons for declining.** Results from completed SiCET that were answered twice.

Of the 124 families who were invited to participate in the study, 86 (69%) accepted (Figure 2). Socio-demographic data of the families (Table 2) showed a higher mean age (p = 0.002) and significantly more mothers with a higher level of education in participating families compared to non-participating families (p = 0.028). No other significant differences in background variables were found between the groups. Reasons given for not participating are shown in Figure 2. Sixteen per cent (n = 14) of the families who answered the SiCET at base-line left the study during the intervention (Figure 2).

**Table 2:** Socio-demographic data of participant and non-participant families in the study

**Characteristic**	**Participants**		**Non- participants**	
	**(n = 86)**		**(n = 38)**	
Age (year) median (range)	32	(20–43)	27	(21–44)
Education	**n**	**(%)**	**n**	**(%)**
Compulsory school	27	(31)	18	(49)
Secondary School	52	(60)	20	(51)
University	7	(8)	0	(0)
Country of birth				
Sweden	51	(59)	22	(58)
Other than Sweden	35	(41)	16	(42)
Marital status				
Single	8	(9)	4	(11)
Married/cohabitant	78	(91)	34	(89)
Occupation				
Studying	8	(9)	3	(8)
Working	57	(66)	27	(71)
Other*	21	(25)	8	(21)

*unemployed, temporary disability leave.

### Results retrieved from the first SiCET, related to the socio-demographic data of parents

The median age of children when the families were recruited was 2 months (range: new-born to 4 years), 55% of whom were recruited when children were less than one month old. The prevalence of smoking mothers and fathers was 48% and 72%, respectively. Of the parents of non-Swedish origin, 17% (n = 5) of mothers and all fathers (n = 25) were smokers. Among all smokers, 39% answered they were indoor and 61% only outdoor smokers.

Eighty-four per cent (n = 72) of the families who answered the SiCET at base-line stated a “willingness to change behaviour”. Significantly more Swedish-born mothers wanted to make changes than foreign born mothers (p = 0.019). No such difference was found among fathers. All parents who received a referral to an expert in smoking cessation support (n = 19) stated a willingness to change their behaviour.

Results from log-books described that the nurses recommended “quit smoking” websites to 51% of families, used motivational interviewing (MI) with 64% of families, and handed out booklets about smoking to 49% of families. The nurses reported using interpreters in meetings with six families, either face to face or via the telephone. Some parents did not want to use an interpreter when they were offered.

### Results retrieved from the SiCET at follow up related to the socio-demographic data of parents

Seventy-two (84%) of the participating families answered the SiCET at follow up (Figure 2). Fifty-five of the seventy-two families (76%) were only smoking outdoors or had stopped smoking at follow up (Table 3). The total number of outdoor-only smokers was unchanged (base-line 60%, n = 43, follow up 63%, n = 45). Nine families (13%) changed from indoor to outdoor smoking and seven families (10%) changed from outdoor to indoor smoking. The outdoor to indoor group mixed their behaviour and were also smoking outdoors with the door closed (n = 6).

**Table 3:** All implemented actions related to changes and lack of changes (n = families)

	**Quit smoking n = 9**	**Reduced cigarette consumption n = 32**	**From indoor to outdoor smokers n = 9**	**Outdoor smokers only n = 36**	**Indoor smokers n = 18**	**From outdoor to indoor smokers n = 7**
**Actions**						
Number of visits to CHC ≥6	4	24	5	21	11	5
Collaboration with ANC*	7	19	4	24	14	4
Recommend websites	4	20	7	18	9	3
Booklets/written information	6	18	8	22	9	3
Home visits	5	23	4	28	12	5
Motivational interviewing	8	27	9	31	16	5
**Measurements**						
Willingness to change**	9	27	7	32	13	5
Cotinine/urine ≤6***	7	17	5	26	8	4
**Socio-demographic data**						
Single parent	1	2	2	3	3	0
Education ≤ 12 years	7	21	6	24	15	3
Born outside Sweden	2	12	3	7	7	7
Unemployed/studying/temporary disability leave	2	5	3	4	4	2

*ANC = Antenatal Care.

**From SiCET answered the first time: the question about willingness to change.

*** Urine sample number 2.

According to the log books, the participating families visited their CHC centre on average six times during the intervention period (md; range 1 to14). Significantly more home visits (67%) were made to families where the child was <1 month of age compared to >1 month (p = 0.013). No differences regarding home visits were found between parents born in Sweden or foreign born parents. No extra home visits were performed by nurses because of the project.

In the follow up SiCET there were no differences found in the willingness to change behaviour among the mothers (p = 0.573) or the fathers (p = 0.582) in relation to their answers at base-line. The results showed that nurses used MI and handed out written information on smoking more often to Swedish-born parents than to foreign born parents (p = 0.001). The logistic regression didn’t show any associations between nurses’ actions as registered in the log-books (MI, booklets, websites, collaboration with antenatal care, referrals to a certified tobacco treatment specialist) the outcome of the follow-up SiCET, and parents’ socio-demographic data. However, groups were small and actual differences might not have been revealed.

#### Parents who quit smoking

During the intervention period, 19 referrals (Swedish-born n = 16) were sent to a tobacco treatment specialist for smoking cessation support. Two of the nineteen referred parents, both foreign born, quit smoking. An additional eight parents (from the quit smoking group n = 10) quit smoking by themselves with no extra help from a certified tobacco treatment specialist. Eight of the 10 parents who reported that they had quit did so, weeks or months after they had filled in the SiCET at follow up, when nurses contacted them to give the results of their child’s second urine test (Table 3).

#### Families reducing cigarette consumption

The number of cigarettes smoked in the home was reduced in 32 families (44%). Twenty-seven of these families had reported a willingness to change behaviour and the nurses used MI with all these families (Table 3). Nineteen families smoked outdoors and 13 indoors.

#### Families who changed from indoor to outdoor smoking

Nine of families who were indoor smokers at base-line (n = 25) changed to be outdoor smokers at follow up. Six of these nine families were Swedish-born. CHC-nurses’ use of motivational interviewing, reference to websites and providing of booklets were more frequent in the group who made these changes than in the group that changed from outdoor to indoor smoking (Table 3). One child in the indoor to outdoor group showed a higher urine cotinine level which may be explained by the child having a smoking grandmother whom the family often visited.

#### Families who changed from outdoor to indoor smoking

Ten per cent of families (n = 7) who were outdoor smokers at base-line changed to be indoor smokers at follow up. The parents in this group were all born outside Sweden. CHC-nurses less frequently informed this group of websites, used motivational interviewing and provided booklets than in the group classified as ‘indoor to outdoor smoking’ (Table 3). Three of the children in the ‘outdoor to indoor smoking group’ (n = 7) had cotinine levels in their urine of <6 ng/ml both at base-line and follow up.

#### Families’ indoor smoking

Thirteen of the 20 families with indoor smokers were Swedish-born. Eight of 20 urine samples among the children in this group showed a decrease of cotinine levels during the intervention and some of them went from high levels to low levels (254 to 12 ng/ml). Some of the parents smoked fewer cigarettes and/or had changed smoking behaviour to include more outdoor than indoor smoking (Table 3). Four children had cotinine levels of <6 ng/ml both at base-line and follow-up and six had decreased levels from >6 ng/ml to <6 ng/ml. Five children had decreased levels, but were still >6 ng/ml, and four children had increased levels from base-line to follow up. One child did not provide a follow up urine test.

#### Families’ outdoor smoking

Twenty-nine of 36 families (81%) who only smoked outdoors were Swedish born. Stricter outdoor smoking policies and fewer cigarettes per day were observed in this group during the intervention (Table 3).

### Cotinine levels in urine

Results of measurement of cotinine levels in urine at base-line and follow up are shown in Table 4. The proportion of children with values of <6 ng/ml increased by 25% (p = 0.05) and the proportion with values of >6 ng/ml decreased by 36% from base-line to follow up. Two of the children (whose parents were outdoor smokers only) were twins and showed almost identical cotinine levels to each other both at base-line and follow up. In some cases where parents quit smoking or changed from indoor to outdoor smoking, the children still had cotinine levels of >6 ng/ml. These children had grandparents who were smokers whom they often met. In the indoor smokers group some of the parents smoked both indoors and outdoors and began smoking more often with an open door or window when smoking indoors.

**Table 4:** Results of cotinine in urine before and after the intervention

**Outcome groups**	**Base-line value**		**Follow-up value**		**Drop-outs**
	**n = 73**		**n = 73**		
					**n = 9**
	<6 ng/ml**	**>6 ng/ml**	<6 ng/ml**	**>6 ng/ml**	
Quit smoking (n = 9)	6	3	8	1*	
From indoor smoking to outdoor smoking (n = 9)	5	1	5	1*	3
Indoor smoking only (n = 20)	6	13	11	8	1
From outdoor smoking to indoor smoking (n = 7)	4	2	4	2	1
Outdoor smoking only (n = 37)	22	11	26	7	4
Summary	43	30	54	19	9

*The children often meet smoking grandparents.

A value <6 ng/ml was estimated as the limit of non-measurable ETS exposure.

Six of the children had cotinine levels between 125 and 255 ng/ml at base-line and all of them were breast-fed children of smoking mothers. At follow up the mothers had stopped breast-feeding and the children’s values were lowered to between six and 49 ng/ml. These smoking mothers were both indoor and outdoor smokers at follow up.

### Results from the nurses

Seven of the nurses (30%) who took part in the intervention had overall successful results in their area. The combined results from these groups showed a decrease of smokers in families when the child was 8 months of age, from 20% in 2009 to 12% in 2011 as shown in Table 5 together with the national and regional data for comparison. However, there was a big difference between the rest of the nurses, according to their ability to recruit families and support them in their behaviour change.

**Table 5:** Data from seven nurses (30%) who participated in the intervention with the greatest improvement of results in their Child Health Care areas, are shown as A to G

	**CHCs annual statistics in 2009**			**CHCs annual statistics in 2011**		
**CHC area**	Children 8 months n*	**Smokers in the family%**	Smokers in the family n*	Children 8 months n*	**Smokers in the family%**	Smokers in the family n*
A	28	21.4	6	60	3.4	2
B	28	14	4	47	10.6	5
C	37	16.2	6	52	13.5	7
D	66	19.6	13	45	17.7	8
E	44	25	11	53	13	7
F	53	17	9	52	8	4
G	47	22.7	11	64	15.6	10
Total in the areas	303	20	60	373	12	43
Total in the county		15.5			14.6	
Total in the country		13.7			13.6	

* = number of

**Comparative data from regional and national levels are shown for comparison of smokers in the family when child is 8 months old at base-line in 2009 and follow-up in 2011.

## Discussion

The focus of this study was on the protection of children from tobacco smoke exposure by using a bundle of evidence based actions in an improvement project. The main result is that there are some indications of decreased ETS exposure for children in the families who had CHC nurses who participated in the improvement project. Of the families in the intervention 69% succeeded in their ambitions to increase the protection of their child/children from ETS exposure in different ways with the support from CHC nurses. In addition, one third of the nurses reported successful results overall in their area, compared to results on a national level.

One central component in the bundle was the SiCET questionnaire. It seems to provide a helpful basis for dialogue with parents who are smokers [34]. One of the questions in the SICET which addresses parents’ willingness to change their behaviour in order to protect their child from ETS exposure was the starting point for the dialogue. The answers to this question provide nurses an opportunity to use MI to discuss changes in smoking behaviour. The use of MI as an approach in dialogue with parents gives nurses the possibility to reinforce change talk and has been advocated in conversations about changing life habits [32,46] and has been shown to be effective in supporting smoking cessation [28]. The CHC nurses in this study supported the parents’ belief in themselves by showing confidence in their ability to carry out the changes they wished to make.

In order to customize interventions to better fit the needs of different groups, a bundle of actions was compiled in this intervention, instead of conducting further tests of one evidence-based intervention at a time [31]. A further reduction in smoking among parents in Sweden has been difficult to achieve especially among some groups in the population [16]. There are still barriers to overcome. Blackburn et al. [1] reported, in their cross-sectional survey of UK families that even if 86% of the families knew the adverse health effects of ETS exposure in children, over 80% of these families continued to smoke in their homes. Qualitative studies have shown that the reason why disadvantaged mothers continued to smoke in the home was that they have to deal with the tension between ‘coping’ and ‘caring’ [47,48]. In this study, 69% of the families which participated in the intervention succeeded in their ambitions to increase the protection of their child/children from ETS exposure in different ways; by changing smoking behaviour, smoking less or quitting smoking.

In the group of successful nurses (30%) the reduction of smokers in the families when child was 8 months old, 8%, can be compared to the figures for the county as a whole where the reduction was low during the same period i.e. from 15.5% in 2009 to 14.6% in 2011. No reduction of smoking in families with children aged 8 months was found in the country as a whole. Corresponding figure for the whole country were 13.7 and 13.6 during the same period (Table 5). The latter results were based on the CHC’s annual data from the National Board of Health and Welfare’s report in 2012. The findings are in accordance with other studies which demonstrate the importance of helping parents to develop strategies other than smoking cessation to protect children from ETS exposure in their homes [49]. For example, a community-based intervention study from Portugal on how to make homes smoke-free showed a 10% decrease in ETS exposure in primary school children [6]. The British community-based intervention ‘smoke-free homes’ delivered through schools, health care settings and community events increased smoke-free homes from 35% to 68%, six months after the intervention in an area with low socio-economic status. The study was based on self-reporting. The improvements were gained despite that no parent reported that they quit smoking [5]. The results in this study, both from self-reports and to some extent cotinine levels in urine, indicate that nurses’ actions have influenced parents and their willingness and ability to make changes in order to protect their child from ETS exposure even if they are not motivated to quit smoking, findings which are in concordance with other studies [1,2].

All of the ten participating parents who quit smoking had expressed willingness to change their behaviour for the sake of their children in the beginning of the study. Eight quit with the only support from the CHC nurses, thus without any support from smoking cessation professionals. The nurses’ use of MI in combination with the SiCET may have provided the necessary support for the parents’ self-efficacy by helping them believe in themselves and become confident enough to quit smoking [50].

Providing nurses with the ability to refer parents to a certified tobacco treatment specialist was made in order to make it possible for them to focus on assisting parents in creating smoke-free practices for the home, without spending time on the quitting process. The possibility for the nurses to primarily focus on parents in their ambitions also strengthened the parents to protect their child from ETS exposure when smoking friends and relatives visited their homes.

The SiCET is a questionnaire which provides a comprehensive picture of the child’s ETS exposure [34]. Although the questionnaire is self-reported, it intends to be a support in the dialogue with the parents. The SiCET was used in combination with tests of children’s cotinine levels in urine. Parents have been shown to have a positive attitude to the cotinine tests during the child’s health care visits, whether if they were smokers or not [51]. In this study, urine samples were analysed and compared before and after the intervention to demonstrate to the parents if their behaviour changes could be seen through this objective measurement of their child’s ETS exposure. The results of urine cotinine analyses in combination with the SiCET gave the nurses a possibility to have a more detailed dialogue with the parents especially in cases where cotinine values were inconsistent with the answers in the SiCET. One finding in such a dialogue was high cotinine levels in breast-fed children whose mothers smoked. This finding is in accordance with other studies showing five to ten times higher concentrations of cotinine among breast-fed children of smoking mothers compared to bottle fed children [52].

According to Swedish standard practice for CHC nurses, home visits to families with a new-born are recommended. This study showed that nurses primarily visited families in their homes when the child was new-born. Home visits have a preventive effect in families where children are at risk of poor social home conditions which may affect their health in a negative way [53]. An international comparison has shown that well-child care in Denmark and in England have a stronger emphasis on home-visits in their system [54]. The need of more selective actions among families with special needs besides the general approach has been pointed out in a Swedish study [14]. More frequent home visits to socially disadvantaged families might contribute to more successful tobacco prevention.

The positive results of protecting children from tobacco smoke achieved in this study cannot be attributed to one single intervention, but rather the combination of the interventions in the bundle. The mode of implementation and testing of the intervention bundle through collaborative learning has been shown effective in other quality improvement projects [26,27]. The educational activities of the nurses were combined with actions that have been shown to increase chances for sustainable improvements [55]. However, even if positive effects were shown, there was a large variation in the adherence to the bundle between different CHCs and individual nurses. All nurses used the SiCET but other activities in the bundle were used to a varying extent and are not yet provided in a systematic way. The model for improvement thus needs to be further developed and evaluated in order to enhance further improvement and sustainability of the results. A recently presented coaching model for improvement teams may be one helpful addition [Godfrey MM, Andersson-Gare B, Nelson EC, Nilsson M, Ahlstrom G: *Coaching interprofessional teams in health care improvement,* submitted].

Although the collaborative learning sessions had one of its focus on reaching foreign born parents, the nurses in this study used MI less often to this group of parents than to Swedish born parents. One reason could be linguistic problems but not the only [56]. Few nurses used interpreters in dialogue with parents, and some parents chose not to have interpreters involved when offered. Migrants’ perception of using interpreters in health care is that they can be impeding in terms of insecure literal translation, create a feeling of dependency, and uncertainty about confidentiality [57]. On the other hand, interpreters can facilitate communication if they work as communication aids and are respectful, keeping the code of confidentiality and have a professional attitude [57]. Interpreters in health care have proven to be underused and dependent on the individual health-care practitioner’s own initiative and knowledge according to other studies [58]. Subtleties in language mean that an interpreter is needed to limit misunderstandings and are thus crucial to maintain a high standard of health care [59,60]. To our knowledge there are no studies using MI through interpreters. A further opportunity for improvement would thus be a study in how to more systematically use interpreters in combination with MI.

Further, the foreign born parents were not provided with booklets to the same extent as Swedish-born parents, despite that they were available in all the languages used by participating parents in the study. A previous study showed that parents want to have and read information concerning children and their health [21] and migrants want written information both in Swedish and in their native language [57]. Thus booklets written in parents’ native languages may help assist them in their decision to change their smoking behaviour. In addition, parents could use the information to inform relatives and friends as it has been shown that even if parents are non-smokers, grandparents may be smokers and need to be informed on how to protect their grandchildren from ETS exposure [61]. Why nurses did not use this opportunity equally with all participants was not part of this study. More studies are needed to understand how to reinforce the use of the bundle of interventions in order to also reach foreign born parents.

Future larger evaluative studies, carried out in different contexts can be helpful in providing more knowledge on which combinations of interventions are most efficient in different circumstances [62]. The impact of using the collaborative learning approach in this kind of intervention also needs to be further explored with in depth qualitative studies. Some of the nurses seem to have been very successful in changing their traditional way of working while others, just like smoking parents seem to have more difficulties in changing their habits.

### Study limitations

The final number of families who participated in the intervention resulted in low numbers in each sub-group which limited the opportunity to reach statistical significance in some of the analysis. Despite intense efforts, it was difficult to recruit a large number of nurses to take part in the intervention due to high workloads in the CHC areas. More nurses would most likely have been able to recruit more parents to the project. Furthermore, the nurses’ engagement and use of the suggested actions also had a role in the results which was evident from differences in the amount of data in the log-books. The agreed actions were not used systematically by the nurses among all parents. An evaluation of the nurses’ changed behaviour would have been interesting and important, but the methodology used in this intervention was not designed for this purpose.

The short follow-up period is a limitation in the study as sustainability of smoking cessation needs to be followed over time. Another limitation is the lack of a control group, but the positive change over time, before and after the improvement project regarding children’s ETS exposure in the studied areas, also in relation to the county and the country as a whole, indicates a positive effect. Further comparisons will be provided with matched control areas from another county in a larger future study.

## Conclusion

To reduce children’s exposure to ETS seems to be possible even in areas with a large proportion of smoking parents, through the support from CHC nurses who use a bundle of evidence based interventions. However, the implementation of new working models according to such a bundle is difficult. The collaborative learning applied in this study has resulted in a more evidence based practice among some CHC nurses, while others did not seem to have changed their working habits. Further exploration on how to strengthen the improvement model is thus needed.

## Competing interests

The authors declare that they have no competing interests.

## Authors’ contribution

NC, AKJ and BAG designed the study. NC organized and contributed to the learning sessions and supported the nurses during the intervention period. NC collected data. NC, AKJ, BAG and AA contributed to the evaluation and manuscript preparation. All authors read and approved the final manuscript.

## Pre-publication history

The pre-publication history for this paper can be accessed here:

http://www.biomedcentral.com/1471-2431/13/76/prepub

## References

[B1] Blackburn C Spencer N Bonas S Coe C Dolan A Moy R Effect of strategies to reduce exposure of infants to environmental tobacco smoke in the home: cross sectional survey BMJ 2003 327 7409 257 10.1136/bmj.327.7409.257 12896936 PMC167160

[B2] Johansson A Hermansson G Ludvigsson J How should parents protect their children from environmental tobacco-smoke exposure in the home? Pediatrics 2004 113 4 e291 295 10.1542/peds.113.4.e291 15060255

[B3] Ding D Wahlgren DR Liles S Matt GE Oliver M Jones JA Hovell MF A second reporter matters: agreement between parents’ and children’s reports of smoking bans in families Am J Prev Med 2011 40 5 572 575 10.1016/j.amepre.2010.12.020 21496758 PMC3107008

[B4] Priest N Roseby R Waters E Polnay A Campbell R Spencer N Webster P Ferguson-Thorne G Family and carer smoking control programmes for reducing children’s exposure to environmental tobacco smoke Cochrane Database Syst Rev 2008 4 CD001746 10.1002/14651858.CD001746.pub2 18843622

[B5] Alwan N Siddiqi K Thomson H Lane J Cameron I Can a community-based ‘smoke-free homes’ intervention persuade families to apply smoking restrictions at homes? J Public Health (Oxf) 2011 33 1 48 54 10.1093/pubmed/fdq073 20930040

[B6] Precioso J Samorinha C Calheiros JM Macedo M Antunes H Campos H Second hand smoke (SHS) exposure in children. An evaluation of a preventative measure Rev Port Pneumol 2010 16 1 57 72 20054508

[B7] Edvardsson K Garvare R Ivarsson A Eurenius E Mogren I Nystrom ME Sustainable practice change: professionals’ experiences with a multisectoral child health promotion programme in Sweden BMC Health Serv Res 2011 11 61 10.1186/1472-6963-11-61 21426583 PMC3077331

[B8] Crone MR Verlaan M Willemsen MC van Soelen P Reijneveld SA Sing RA Paulussen TG Sustainability of the prevention of passive infant smoking within well-baby clinics Health Educ Behav 2006 33 2 178 196 10.1177/1090198105276296 16531512

[B9] Chen X Stanton B Hopper J Khankari N Sources, locations, and predictors of environmental tobacco smoke exposure among young children from inner-city families J Pediatr Health Care 2011 25 6 365 372 10.1016/j.pedhc.2010.04.014 22018427

[B10] Mangrio E Hansen K Lindstrom M Kohler M Rosvall M Maternal educational level, parental preventive behavior, risk behavior, social support and medical care consumption in 8-month-old children in Malmo Sweden BMC Publ Health 2011 11 1 891 10.1186/1471-2458-11-891 PMC3280332 22114765

[B11] Wallby T Hjern A Parental region of birth, socio-economic status and infants’ exposure to second-hand smoke Acta Paediatr 2008 97 11 1542 1545 10.1111/j.1651-2227.2008.00964.x 18702638

[B12] Bolte G Fromme H Socioeconomic determinants of children’s environmental tobacco smoke exposure and family’s home smoking policy Eur J Public Health 2009 19 1 52 58 19033356 10.1093/eurpub/ckn114

[B13] Laaksonen M Rahkonen O Karvonen S Lahelma E Socioeconomic status and smoking: analysing inequalities with multiple indicators Eur J Public Health 2005 15 3 262 269 10.1093/eurpub/cki115 15755781

[B14] Wallby T Hjern A Child health care uptake among low-income and immigrant families in a Swedish county Acta Paediatr 2011 100 11 1495 1503 10.1111/j.1651-2227.2011.02344.x 21535134

[B15] Berlin A Johansson SE Tornkvist L Working conditions and cultural competence when interacting with children and parents of foreign origin–primary child health nurses’ opinions Scand J Caring Sci 2006 20 2 160 168 10.1111/j.1471-6712.2006.00393.x 16756521

[B16] Developement of smoking and socioeconomic differences *(Rökningens utveckling och socioekonomiska skillnader)*; http://www.fhi.se/Aktuellt/Nyheter/Rokningens-utveckling-och-socioekonomiska-skillnader/

[B17] Polanska K Hanke W Ronchetti R van den Hazel P Zuurbier M Koppe JG Bartonova A Environmental tobacco smoke exposure and children’s health Acta Paediatr Suppl 2006 95 453 86 92 17000575 10.1080/08035320600886562

[B18] Goksor E Amark M Alm B Gustafsson PM Wennergren G The impact of pre- and post-natal smoke exposure on future asthma and bronchial hyper-responsiveness Acta Paediatr 2007 96 7 1030 1035 10.1111/j.1651-2227.2007.00296.x 17498194

[B19] Breast feeding and smoking behaviour among parents - Children born 2007 *Amning och föräldrars rökvanor - barn födda 2007)*; http://www.socialstyrelsen.se/publikationer2009/2009-10-115 (In Swedish

[B20] Carlsson N Johansson A Hermansson G Andersson-Gare B Child health nurses’ roles and attitudes in reducing children’s tobacco smoke exposure J Clin Nurs 2010 19 3–4 507 516 19686317 10.1111/j.1365-2702.2009.02847.x

[B21] Carlsson N Johansson A Hermansson G Andersson-Gare B Parents’ attitudes to smoking and passive smoking and their experience of the tobacco preventive work in child health care J Child Health Care 2011 15 4 272 286 10.1177/1367493510382243 21078698

[B22] Crossing the quality chasm: a New health system for the 21st centuryhttp://www.nap.edu/openbook.php?isbn=0309072808 25057539

[B23] Bero LA Grilli R Grimshaw JM Harvey E Oxman AD Thomson MA Getting research findings into practice: closing the gap between research and practice: an overview of systematic reviews of interventions to promote the implementation of research findings BMJ: British Medical Journal 1998 317 7156 465 10.1136/bmj.317.7156.465 9703533 PMC1113716

[B24] Open comparisons (Öppna jämförelser) http://www.skl.se/vi_arbetar_med/oppnajamforelser/halso-_och_sjukvard_2/ojhs201023682628

[B25] Langely GJ Moen RD Nolan KM Nolan TW Norman CL Provost LP The improvement guide. A practical approach to enhancing organizational performance 2009 San Fransisco: Jossey-Bass

[B26] Peterson A Carlhed R Lindahl B Linström G Åberg C Andersson Gare B Bojestig M Improving guidline adherence through intensive quality improvement and the Use of a national quality register in sweden for acute myocardial infarction Qual Manag Health Care 2007 16 1 25 37 17235249 10.1097/00019514-200701000-00005

[B27] Johnson JK Barach PR Quality improvement methods to study and improve the process and outcomes of pediatric cardiac care Prog Pediatr Cardiol 2011 32 2 147 153 10.1016/j.ppedcard.2011.10.014

[B28] Hettema JE Hendricks PS Motivational interviewing for smoking cessation: a meta-analytic review J Consult Clin Psychol 2010 78 6 868 884 21114344 10.1037/a0021498

[B29] Hurt RD Ebbert JO Hays JT McFadden DD Treating tobacco dependence in a medical setting CA Cancer J Clin 2009 59 5 314 326 10.3322/caac.20031 19706827 PMC3903579

[B30] Ellström P-E Eklund J Kock H Lindström L Melin U Nowledge creation through collaborative research: an emerging model 1999 Falun; Linköping: HSS-99 Conference, Falun; CMTO Research Papers, CMTO Linköping University

[B31] Dawson D Endacott R Implementing quality initiatives using a bundled approach Intensive Crit Care Nurs 2011 27 3 117 120 10.1016/j.iccn.2011.03.006 21511476

[B32] Soderlund LL Madson MB Rubak S Nilsen P A systematic review of motivational interviewing training for general health care practitioners Patient Educ Couns 2011 84 1 16 26 10.1016/j.pec.2010.06.025 20667432

[B33] Johansson A Halling A Hermansson G Ludvigsson J Assessment of smoking behaviors in the home and their influence on children’s passive smoking: development of a questionnaire Ann Epidemiol 2005 15 6 453 459 10.1016/j.annepidem.2004.09.012 15967393

[B34] Carlsson N Alehagen S Andersson Gare B Johansson A “Smoking in children’s environment test”: a qualitative study of experiences of a new instrument applied in preventive work in child health care BMC Pediatr 2011 11 1 113 10.1186/1471-2431-11-113 22172056 PMC3267672

[B35] Boldemann C Gilljam H Lund KE Helgason AR Smoking cessation in general practice: the effects of a quitline Nicotine Tob Res 2006 8 6 785 790 10.1080/14622200601004059 17132526

[B36] Tobacco-free children (Tobaksfria barn) http://www.fhi.se/PageFiles/7013/Tobaksfria_barn_webb.pdf (In Swedish)

[B37] Kennedy A Sapsis KF Stokley S Curtis CR Gust D Parental attitudes toward human papillomavirus vaccination: evaluation of an educational intervention, 2008 J Health Commun 2011 16 3 300 313 10.1080/10810730.2010.532296 21161814

[B38] Vogt C Schaefer M Knowledge matters–impact of two types of information brochure on contraceptive knowledge, attitudes and intentions Eur J Contracept Reprod Health Care 2012 17 2 135 143 10.3109/13625187.2011.643837 22200343

[B39] Andersson-Ellström A Mödrahälsovård, sexuell och reproduktiv hälsa 2008 Stockholm: Svensk förening för obstetrik och gynekologi

[B40] Ekström A Widström A Nissen E Does continuity of care by well-trained breastfeeding counselors improve a mother’s perception of support? Birth 2006 33 2 123 130 10.1111/j.0730-7659.2006.00089.x 16732777

[B41] Jansson A Petersson K Uden G Nurses’ first encounters with parents of new-born children–public health nurses’ views of a good meeting J Clin Nurs 2001 10 1 140 151 10.1046/j.1365-2702.2001.00456.x 11820231

[B42] Øvretveit J Bate P Cleary P Cretin S Gustafson D McInnes K McLeod H Molfenter T Plsek P Robert G Quality collaboratives: lessons from research Qual Saf Health Care 2002 11 4 345 351 10.1136/qhc.11.4.345 12468695 PMC1757995

[B43] Nembhard IM Learning and improving in quality improvement collaboratives: which collaborative features do participants value most? Heal Serv Res 2009 44 2p1 359 378 10.1111/j.1475-6773.2008.00923.x PMC2677044 19040423

[B44] Ayers LR Beyea SC Godfrey MM Harper DC Nelson EC Batalden PB Quality improvement learning collaboratives Qual Manag Health Care 2005 14 4 234 247 10.1136/qshc.2004.011924 16227872

[B45] Benowitz NL Cotinine as a biomarker of environmental tobacco smoke exposure Epidemiol Rev 1996 18 2 188 204 10.1093/oxfordjournals.epirev.a017925 9021312

[B46] Rubak S Sandbaek A Lauritzen T Christensen B Motivational interviewing: a systematic review and meta-analysis Br J Gen Pract 2005 55 513 305 312 15826439 PMC1463134

[B47] Robinson J Kirkcaldy AJ Disadvantaged mothers, young children and smoking in the home: mothers’ use of space within their homes Health Place 2007 13 4 894 903 10.1016/j.healthplace.2007.03.001 17499542

[B48] Robinson J Kirkcaldy AJ ‘You think that I’m smoking and they’re not’: why mothers still smoke in the home Soc Sci Med 2007 65 4 641 652 10.1016/j.socscimed.2007.03.048 17482738

[B49] Rosen LJ Noach MB Winickoff JP Hovell MF Parental smoking cessation to protect young children:a systematic review and meta-analysis Pediatrics 2012 129 1 141 152 10.1542/peds.2010-3209 22201152

[B50] Strecher VJ Bauman KE Boat B Fowler MG Greenberg R Stedman H The role of outcome and efficacy expectations in an intervention designed to reduce infants’ exposure to environmental tobacco smoke Health Educ Res 1993 8 1 137 143 10.1093/her/8.1.137 11067181

[B51] Winickoff JP Tanski SE McMillen RC Ross KM Lipstein EA Hipple BJ Friebely J Klein JD Acceptability of testing children for tobacco-smoke exposure: a national parent survey Pediatrics 2011 127 4 628 634 10.1542/peds.2010-2462 21422089 PMC3387887

[B52] Becker AB Manfreda J Ferguson AC Dimich-Ward H Watson WT Chan-Yeung M Breast-feeding and environmental tobacco smoke exposure Arch Pediatr Adolesc Med 1999 153 7 689 691 10401801 10.1001/archpedi.153.7.689

[B53] Donovan EF Ammerman RT Besl J Atherton H Khoury JC Altaye M Putnam FW Van Ginkel JB Intensive home visiting is associated with decreased risk of infant death Pediatrics 2007 119 6 1145 1151 10.1542/peds.2006-2411 17545382

[B54] Kuo AA Inkelas M Lotstein DS Samson KM Schor EL Halfon N Rethinking well-child care in the United States: an international comparison Pediatrics 2006 118 4 1692 1702 10.1542/peds.2006-0620 17015563

[B55] Greenhalgh T Robert G Bate P Macfarlane F Kyriakidou O Diffusion of innovations in health service organisations 2005 Malden, Massachusetts: Blackwell Publishing Ltd

[B56] Berlin A Tornkvist L Hylander I Watchfully checking rapport with the primary child health care nurses - a theoretical model from the perspective of parents of foreign origin BMC Nurs 2010 9 14 10.1186/1472-6955-9-14 20646287 PMC2918611

[B57] Hadziabdic E Heikkila K Albin B Hjelm K Migrants’ perceptions of using interpreters in health care Int Nurs Rev 2009 56 4 461 469 10.1111/j.1466-7657.2009.00738.x 19930075

[B58] Kale E Syed HR Language barriers and the use of interpreters in the public health services. A questionnaire-based survey Patient Educ Couns 2010 81 2 187 191 10.1016/j.pec.2010.05.002 20542656

[B59] Flores G The impact of medical interpreter services on the quality of health care: a systematic review Med Care Res Rev 2005 62 3 255 299 10.1177/1077558705275416 15894705

[B60] The Swedish health and medical services act. (Hälso- och sjukvårdslagen) (Hälso- och sjukvårdslagen); http://www.notisum.se/rnp/sls/lag/19820763.htm (In Swedish)

[B61] Yousey Y Family attitudes about tobacco smoke exposure of young children at home MCN Am J Matern Child Nurs 2007 32 3 178 183 10.1097/01.NMC.0000269568.17432.72 17479055

[B62] Walshe K Understanding what works–and why–in quality improvement: the need for theory-driven evaluation Int J Qual Health Care 2007 19 2 57 59 10.1093/intqhc/mzm004 17337518

